# Neonatal congenital heart surgery: contemporary outcomes and risk profile

**DOI:** 10.1186/s13019-022-01830-w

**Published:** 2022-04-20

**Authors:** Ahmed Abdelrahman Elassal, Osman Osama AL-Radi, Ragab Shehata Debis, Zaher Faisal Zaher, Gaser Abdelmohsen Abdelmohsen, Mazen Shamsaldeen Faden, Nada Ahmed Noaman, Ahmed Ragab Elakaby, Mohamed Esam Abdelmotaleb, Ahmed Mostafa Abdulgawad, Mohamed Saleh Elhudairy, Abdulla Husain Jabbad, Ahmed Abdelaziz Ismail, Norah Bakheet Aljohani, Arwa Mohammed Alghamdi, Ahmed Mohamed Dohain

**Affiliations:** 1 Cardiac Surgery Unit, Department of Surgery, King Abdulaziz University, Jeddah, 21589 Saudi Arabia; 2 Cardiothoracic Surgery Department, Zagazig University, Zagazig, Egypt; 3 Cardiothoracic Surgery Department, Al-Azhar University, Cairo, Egypt; 4 Department of Pediatric Cardiology, King Abdulaziz University, Jeddah, Saudi Arabia; 5 Pediatric Cardiology Division, Department of Pediatrics, Cairo University, Cairo, Egypt; 6 Department of Anesthesia and Critical Care, King Abdulaziz University, Jeddah, Saudi Arabia; 7 Pediatrics Department, Faculty of Medicine (Boys), Al-Azhar University, Cairo, Egypt; 8 Pediatric Cardiac Intensive Care Unit, King Abdulaziz University, Jeddah, Saudi Arabia; 9 Sixth Grade, Faculty of Human Medicine, King Abdulaziz University, Jeddah, Saudi Arabia; 10 Department of Anesthesia and Pain Management, Faculty of Medicine, Cairo University, Cairo, Egypt; 11 Cardiac Surgery Unit, Patient Coordination Unit, King Abdulaziz University, Jeddah, Saudi Arabia

**Keywords:** Congenital heart surgery, Neonatal, Outcome, Risk

## Abstract

**Objective:**

Many studies still dispute the identification of independent risk factors that influence outcome after neonatal cardiac surgery. We present our study to announce the contemporary outcomes and risk profile of neonatal cardiac surgery at our institute.

**Methods:**

We designed a retrospective study of neonatal patients who underwent surgery for congenital heart diseases between June 2011 and April 2020. Demographic, operative, and postoperative data were collected from medical records and surgical databases. The primary outcome was the operative mortality (in-hospital death) and secondary outcomes included hospital length of stay, intensive care unit stay, duration of mechanical ventilation.

**Results:**

In total, 1155 cardiac surgeries in children were identified; of these, 136 (11.8%) were performed in neonates. Arterial switch operations (48 cases) were the most frequent procedures. Postoperatively, 11 (8.1%) patients required extracorporeal membrane oxygenation, and 4 (2.9%) patients had complete heart block. Postoperative in-hospital mortality was 11%. The median postoperative duration of mechanical ventilation, intensive care unit stay, and hospital length of stay were 6, 18, and 24 days, respectively.

**Conclusion:**

The early outcomes of neonatal cardiac surgery are encouraging. The requirement of postoperative extracorporeal membrane oxygenation support, postoperative intracranial hemorrhage, and acute kidney were identified as independent risk factors of mortality following surgery for congenital heart defects in neonates.

## Introduction

Survival after neonatal cardiac surgery has significantly improved as a result of advances in surgical techniques and perioperative management, but significant morbidity persists [1, 2]. Accordingly, trials have shifted from not only improving survival but also reducing morbidity [3]. In surgery for congenital heart diseases, the commonly used outcomes are mortality, duration of mechanical ventilation, intensive care unit (ICU) stay, and hospital stay [4, 5]. However, in neonatal cardiac surgery, the situation is different as the accuracy to measure these outcomes may be questionable because mortality is infrequent, and the other outcomes often have a wide range resulting in the need for a large sample size. Many studies have used surrogate outcome measures, such as low cardiac output syndrome, inotropic score, and vasoactive inotropic score [4–6]. In this study, we sought to identify the risk factors that influence survival after surgery for congenital heart diseases (CHD) in the neonatal population at our institute.

## Materials and methods

### Study population

We conducted a retrospective review of all neonatal patients who underwent cardiac surgery between June 2011 and April 2020 at King Abdulaziz University Hospital in Jeddah, Saudi Arabia. Patients were enrolled if the age at the time of operation was less than 30 days. Our cardiac surgery center only accepts surgical cases from other hospitals, so no case has been accepted by the cardiology team due to clinical reasons or the complexity of the malformations precluding the surgery. The patients' data were collected from hospital medical records and cardiac surgical databases. Approval of the study was obtained from our institutional Research Ethics Board.

### Data collection

Demographic and perioperative data collected included age at surgery, weight, gender, gestational age, birth weight, associated major non-cardiac abnormalities, congenital anomalies, cardiac diagnosis, and preoperative mechanical ventilation. Operative variables included type of operative procedure, cardiopulmonary bypass (CPB) time, aortic cross-clamp time, and deep hypothermic circulatory arrest time (DHCA). The surgical risk stratification was included based on preoperative Risk Adjusted Congenital Heart Surgery (RACHS)-1 score [7]. The primary outcome was the operative mortality (in-hospital death) and secondary outcomes included hospital length of stay, ICU stay, duration of mechanical ventilation. Postoperative data included the requirement of extracorporeal membrane oxygenation (ECMO) support, early postoperative cardiac catheterization intervention, delayed chest closure, diaphragmatic paralysis, chylous effusion, arrhythmias, cerebral infarction, intracranial hemorrhage, limb ischemia, necrotizing enterocolitis (NEC), acute kidney injury (AKI), hepatic injury, and bloodstream infection (sepsis).

### Definitions

Prematurity was defined as birth at < 37 weeks gestation. The operative mortality was defined as death before hospital discharge in the index admission. AKI was defined by an increase in creatinine > 2 times the upper limit of normal (normal range, 0.3–0.7 mg/dL; AKI, > 1.5 mg/dL) [8]. Hepatic injury was defined as aspartate aminotransferase (AST) or alanine aminotransferase (ALT) higher than 2 times the upper limit of normal (normal range, AST 8–40 IU/L; normal range, ALT 7–40 IU/L; hepatic injury, AST, or ALT > 80 IU/L). Elevated AST within the first postoperative 24 h was not considered hepatic dysfunction because AST elevation was likely due to hemolysis related to CPB. Chylous effusion was considered if the triglycerides in the chest tube drain were > 1.25 mmol/L or the patient required medium-chain triglyceride formula. Postoperative positive blood culture was needed to establish bloodstream infection (sepsis).

### Statistical analysis

Statistical analysis was performed using IBM SPSS Statistics for Windows, version 26 (IBM Corp., Armonk, NY, USA). Nominal variables were presented in numbers and percentages, while Numeric variables were presented in the median and interquartile range (IQR) (25th–75th percentiles). The prolonged in-hospital stay was defined if the duration was > 75th percentile of the in-hospital stay duration. Logistic regression analysis was used for the evaluation of mortality predictors. Variables of statistical significance in univariate logistic regression were entered in a multivariate logistic regression model to determine the most important mortality predictors. Comparisons between groups were tested using χ2 and Mann–Whitney U tests for categorical and numeric variables, respectively. Statistical significance was considered if *P* < 0.05.

## Results

### Baseline characteristics

In total, 1155 cardiac surgeries for CHD were identified during the study period; of these, 136 (11.8%) were performed in neonates. Male subjects comprised 64% of the whole study cohort. 17 (12.5%) patients had a gestational age < 37 weeks. The mean age and body weight at surgery were 18 ± 8.0 days and 2.89 ± 0.80 kg, respectively. Mean gestational age was 35.49 ± 4.95 weeks and mean birth weight was 2.82 ± 0.80 kg. 9 (6.6%) patients had associated congenital anomaly apart from their CHD.

**Fig. 1:**
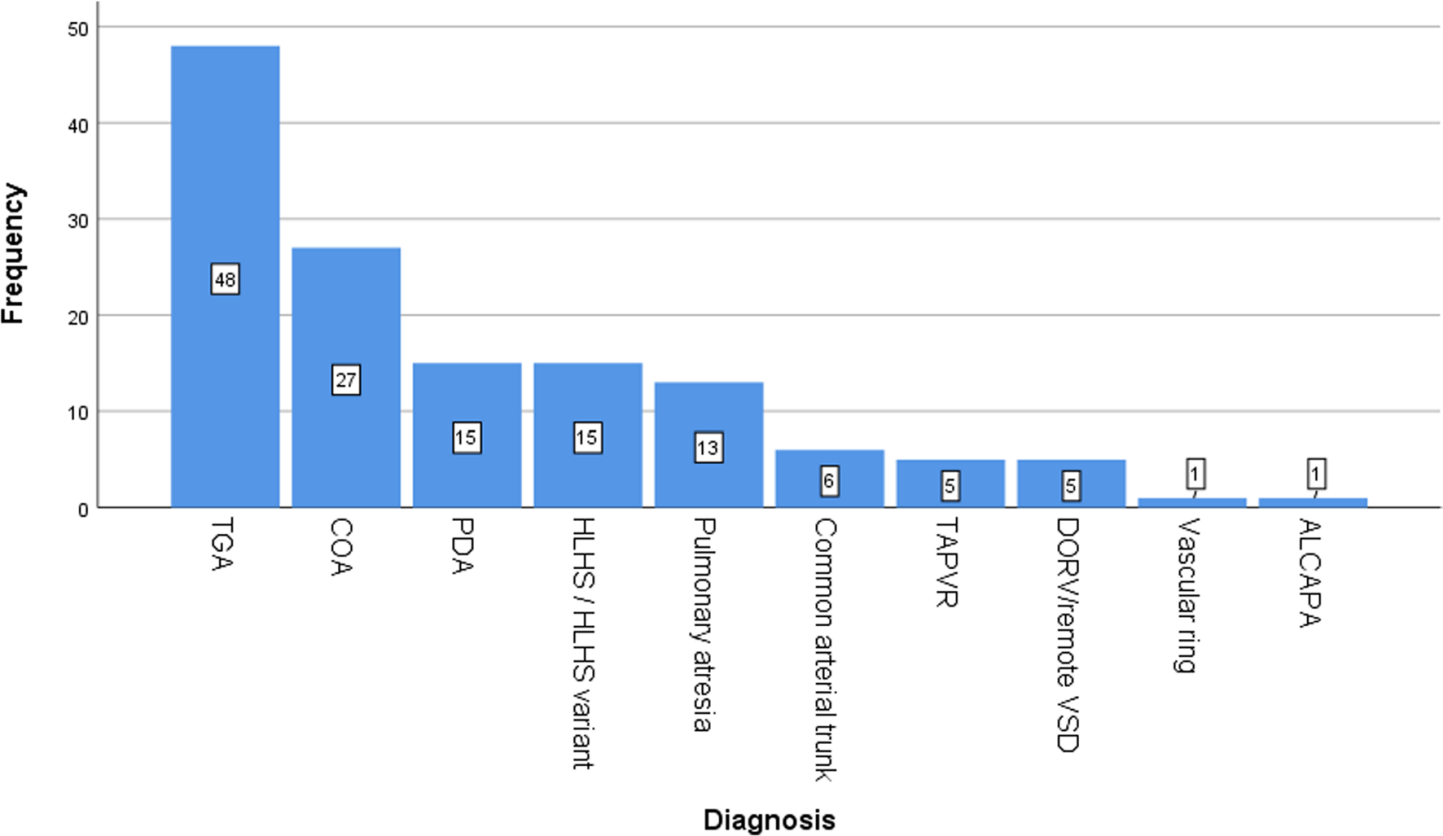
Diagnosis distribution in neonates who underwent cardiac surgery

**Fig. 2:**
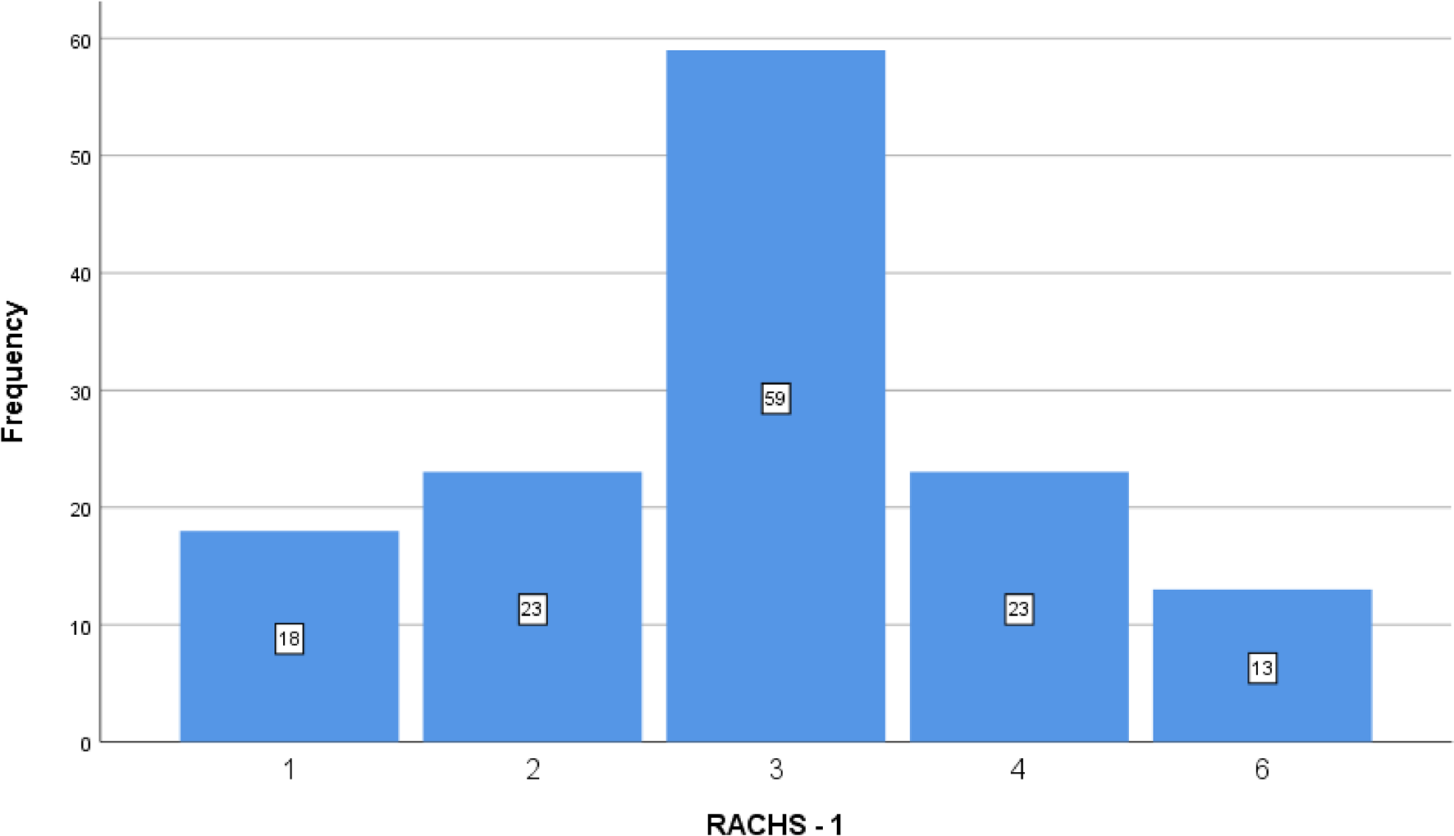
The patients' distribution according to the Risk Adjustment in Congenital Heart Surgery (RACHS-1) scores classification

Transposition of the great arteries (TGA) was the most frequent cardiac diagnosis in the studied patients (35.3%), followed by coarctation of the aorta (CoA; 19.9%), patent ductus arteriosus (PDA; 11%), hypoplastic left heart syndrome (HLHS; 11%), and pulmonary atresia (PA; 9.5%) (Fig. 1). Among the main surgical procedures, arterial switch operations (48 cases) was the most frequent procedure, followed by CoA repair (27 cases), PDA ligation (15 cases), Norwood procedure (15 cases), modified Blalock Taussig shunt (MBTS, 9 cases), common arterial trunk repair (6 cases), total anomalous pulmonary venous return repair (TAPVR; 5 cases), pulmonary artery banding (PAB, 5 cases), vascular ring repair (1 case), and aberrant left coronary artery from the pulmonary artery (ALCAPA, 1 case). The operative RACHS-1 category distribution included 60.3% from categories 3/4 and 9.6% from category 6, Fig. 2. Table 1 displays the demographic and preoperative clinical characteristics of the cohort.

**Table 1:** Characteristics of the studied patients

Parameter	
Age, days	18 ± 8.0
Birth weight, Kg	2.82 ± 0.80
Weight, Kg	2.89 ± 0.80
Gestational age, weeks	35.49 ± 4.95
Male/female, n (%)	87/49( 64/36)
Full term/preterm, n (%)	119/17 (87.5/12.5)
Associated syndromes, n (%)	4 (2.9)
Associated congenital anomalies, n (%)	
Ambiguous genitalia	1 (0.7)
Inguinal hernia	3 (2.2)
Hypospadias	1 (0.7)
Horse show kidney	2 (1.4)
Syndactyly	1 (0.7)
Cutaneous haemangioma	1 (0.7)
Diagnosis, n (%)	
D-TGA	48 (35.3)
Coarctation of aorta	27 (19.9)
PDA	15 (11)
HLHS/ HLHS variants	15 (11)
Pulmonary atresia	13 (9.5)
Common arterial trunk	6 (4.4)
TAPVR	5 (3.7)
DORV- remote VSD/single ventricle	5 (3.7)
Vascular ring	1 (0.7)
ALCAPA	1 (0.7)
RACHS-1, n (%)	
1	18 (13.2)
2	23 (16.9)
3	59 (43.4)
4	23 (16.9)
6	13 (9.6)

D-TGA, Dextro transposition of great arteries; PDA, patent ductus arteriosus; HLH, hypoplastic left heart syndrome; TAPVR, total anomalous pulmonary venous return; DORV, double outlet right ventricle; VSD, ventricular septal defect; ALCAPA, aberrant left coronary artery from the pulmonary artery; RACHS-1, risk adjustment for congenital heart surgery; COA, coarctation of the aorta; MBTS, modified Blalock Taussig shunt

### Perioperative data, postoperative outcome, and morbidity

Preoperative mechanical ventilation was required for 59 (43.3%) patients for a median duration of 4 days. Among the 136 neonatal cardiac surgeries, 110 cases (80.9%) were performed with CPB, and 47 (34.6%) patients underwent DHCA. The median duration of CPB, aortic cross-clamp, and DHCA were 81, 51, 15 min, respectively. The sternum was electively left open after the surgery in 74 (54.4%) patients, and it was closed within a median time interval of 3 days.

Postoperatively, 11 (8.1%) patients required ECMO support, the indications of ECMO conduction were cardiac arrest and ECPR (extracorporeal cardiopulmonary resuscitation) in ICU for 5 cases, failure to wean from cardiopulmonary bypass in the theater room for 4 cases, and low cardiac output in ICU for 2 cases. 4 (2.9%) patients had complete heart block (CHB) necessitating permanent pacemaker (PPM) insertion, 3(2.2%) patients developed junctional ectopic tachycardia (JET), 4 (2.9%) patients needed interventional cardiac catheterization, 16 (11.8%) patients had chylous effusion, and 3 (2.2%) patients had diaphragmatic paralysis. One of the patients needed diaphragmatic plication.

Neurological complications included intracranial hemorrhage in 11 patients (7 of them were on ECMO support) and stroke in 3 (1 patient was on ECMO support). The hepatic injury occurred in 43 (31.6%) patients, 7 (5.1%) developed necrotizing enterocolitis, and 9 (6.6%) patients had AKI (5 patients required peritoneal dialysis). Twenty (14.7%) patients had postoperative bloodstream infections. The commonest organisms were coagulase-negative staphylococci (CONS, 8 cases) followed by acinetobacter (3 cases), klebsiella (3 cases), and pseudomonas (3 cases).

Re-exploration: Four cases were re-operated in the same admission after the initial surgery, three of which were re-explored to control postoperative bleeding and one was re-operated to loosen the pulmonary artery band after a case of coarctation repair plus pulmonary artery banding. Overall, in-hospital mortality after neonatal cardiac surgery was 11%. The median postoperative duration of mechanical ventilation, ICU stay, and hospital length of stay were 6, 18, and 24 days, respectively, Table 2.

**Table 2:** Perioperative and operative data

Parameters	
Preoperative mechanical ventilation, n (%)	59 (43.3)
Preoperative mechanical ventilation, days	4 (2–11)
Bypass, n (%)	110 (80.9)
Bypass time, minutes	81 (60–95)
Aortic cross-clamp time, minutes	51 (38–65)
Circulatory arrest time (minutes)	15 (4–32)
Incision, n (%)	
Median sternotomy	115 (84.6)
Lateral thoracotomy	21 (15.4)
Delayed sternal closure, n (%)	74 (54.4)
Duration of open chest, days	3 (2–5)
Post-operative ECMO support, n (%)	11 (8.1)
Post-operative catheter intervention, n (%)	4 (2.9)
Duration of mechanical ventilation, days	6 (3–11)
ICU stay, days	18 (10–36)
Length of hospital stay, days	24 (15–50)
Post-operative complications, n (%)	
Junctional ectopic tachycardia	3 (2.2)
Complete heart block required PPM	4 (2.9)
Chylothorax	16 (11.8)
Phrenic palsy	3 (2.2)
Cerebral infarction	3 (2.2)
Intracranial hemorrhage	11 (8.1)
Limb ischemia	5 (3.7)
Necrotizing enterocolitis	7 (5.1)
Acute Kidney injury	9 (6.6)
Peritoneal dialysis	5 (3.7)
Elevated Liver enzymes (> 80)	43 (31.6)
Sepsis	20 (14.7)
Mortality, n (%)	15 (11%)

ECMO, extracorporeal membrane oxygenation; ICU, intensive care unit; PPM, permanent pacemaker

### Operative mortality

The requirement of postoperative ECMO support was significantly more frequent among non-survivors compared to the survivors (*P* < 0.0001). The occurrence of postoperative persistent CHB, intracranial hemorrhage, AKI, and peritoneal dialysis was significantly higher in the mortality group compared to the survival group, *p* values were 0.012, < 0.0001, < 0.0001, and 0.035, respectively. There was no statistically significant difference in age at surgery, weight, prematurity, RACHS-1 scores, and need for CPB for both survival and mortality groups, Table 3.

**Table 3:** Comparison between survival and mortality groups

	Survival group (n = 121)	Mortality group (n = 15)	*P* value
Age, days	10 (18–24)	23 (12–30)	0.268
Weight, Kg	3 (2.6–3.4)	3 (2.5 -3.4)	0.856
ICU length of stay, days	18 (11–36)	10 (2–23)	0.022
Hospital length of stay, days	25 (17–50)	17 (9–43)	0.075
Preterm, n (%)	16 (13.2)	1 (7)	0.693
Bypass, n (%)	96 (79.3)	14 (93.3)	0.172
RACHS -1 n (%)			0.625
1&2	38 (31.4)	3 (20)	
3&4	72 (59.5)	10 (66.7)	
6	11 (9.1)	2 (13.3)	
ECMO, n (%)	4 (3.3)	7 (46.7)	< 0.0001
Chylothorax, n (%)	16 (13.2)	0	0.215
CHB required PPM	2 (1.7)	2 (13.3)	0.012
Phrenic palsy, n (%)	2 (1.7)	1 (6.7)	0.298
Sepsis, n (%)	17 (14)	3 (20)	0.463
Intracranial haemorrhage, n (%)	5 (4.1)	6 (40)	< 0.0001
Cerebral infarction, n (%)	3 (2.5)	0	0.537
NEC, n (%)	5 (4.1)	2 (13.3)	0.172
AKI, n (%)	3 (2.5)	6 (40)	< 0.0001
Peritoneal dialysis	3 (2.5)	2 (13.3)	0.035

RACHS-1, risk-adjusted congenital heart surgery; ECMO, extracorporeal membrane oxygenation; CHB, complete heart block; PPM, permanent pacemaker; NEC, necrotizing enterocolitis; AKI, acute kidney injury

#### Univariate analysis of operative mortality

On univariate analysis, there was a significant association between mortality and postoperative ECMO support (*P* < 0.001), persistent CHB (*p* = 0.034), intracranial hemorrhage (*P* < 0.001), and AKI (*P* < 0.001).

There was no statistically significant association of mortality with age at surgery, weight, prematurity, RACHS-1 scores, and other postoperative complications. Table 4 demonstrates a univariate analysis of risk factors for operative mortality.

**Table 4:** Predictors of mortality

	Univariate logistic regression		Multivariate logistic regression	
	OR (95% CI)	*P* value	OR (95% CI)	*P* value
Age, days	0.961 (0.899–1.027)	0.245		
Weight, Kg	0.797 (0.329–1.67)	0.529		
ICU stay	1.01 (0.988–1.030)	0.385		
Preterm	0.469 (0.058–3.812)	0.479		
Bypass	0.274 (0.034–2.18)	0.222		
RACHS-1				
1&2	2.30 (0.341–15.56)	0.392		
3&4	1.3 (0.235–6.78)	0.748		
ECMO	0.039 (0.10–0.163)	< 0.0001	0.147 (0.022–0.983)	0.048
Phrenic palsy	0.235 (0.020–2.764)	0.250		
CHB required PPM	0.109 (0.014–0.842)	0.034	0.157 (0.011–2.22)	0.171
Sepsis	0.654 (0.167–2.561)	0.542		
Intracranial haemorrhage	0.056 (0.016–0.254)	< 0.0001	0.141 (0.025–0.792)	0.026
NEC	0.280 (0.49–1.592)	0.151		
AKI	0.038 (0.008–0.178)	< 0.0001	0.114 (0.015–0.849)	0.034
Peritoneal dialysis	0.165 (0.025–1.082)	0.060		

ICU, intensive care unit; RACHS-1, risk-adjusted congenital heart surgery; ECMO, extracorporeal membrane oxygenation; CHB, complete heart block; PPM, permanent pacemaker; NEC, necrotizing enterocolitis; AKI, acute kidney injury

#### Multivariate analysis of risk factors for operative mortality

Multivariable logistic regression performed using mortality as an outcome and neonatal variables and other risk factors as independent variables, showed the need for postoperative ECMO support [OR (95% CI) 0.147(0.022–0.983), *P* = 0.048], the occurrence of postoperative intracranial hemorrhage [OR (95% CI) 0.141(0.025–0.792), *P* = 0.026], and incidence of AKI [OR (95% CI) 0.114(0.015–0.849), *P* = 0.034] as independent predictors of operative mortality.

Age at surgery, weight, prematurity, RACHS-1 scores, CPB, and other postoperative complications were not found to be independent predictors of operative mortality. Table 4 illustrates multivariate analysis and lists independent risk factors for operative mortality in the whole cohort.

### Postoperative hospital length of stay

Older age at surgery, smaller body weight, lower birth weight, prematurity, higher-complexity operations (RACHS-1), need for CPB, and NEC were associated with prolonged hospital length of stay. On the other hand, prolonged hospital length of stay was not significantly related to ECMO support, CPB time, aortic cross-clamp time, and postoperative complications (apart from NEC), Table 5.

**Table 5:** Comparison between groups regarding the postoperative hospital length of stay

Parameter	Non-prolonged hospital stays (n = 102)	Prolonged hospital stays (n = 34)	*P* value
Age, days	16.5 (9.0 -22.25)	23.5 (16–30)	0.001
Birth weight, Kg	3.0 (2.77- 3.45)	2.35 (0.905–3.00)	< 0.0001
Weight, Kg	3.0 (2.80–3.50)	2.40 (0.99–3.00)	< 0.0001
ICU length of stay, days	14 (8–20)	61.5 (46.0–92.25)	< 0.0001
Preoperative mechanical ventilation duration, days	3.5 (1.0–5.0)	14.0 (4.0–23.0)	0.003
Bypass time, minutes	81.5 (59.20 -96.50)	79.0 (61.50–89.50)	0.461
Cross clamp time, minutes	51.0 (38.50–67.50)	45.0 (28.0–61.0)	0.305
RACHS-1, n (%)			0.001
1&2	23 (22.5)	18 (53)	
3&4	71 (69.7)	11 (32.3)	
6	8 (7.8)	5 (14.7)	
Bypass, n (%)	89 (87.2)	34 (100)	0.001
Preterm, n (%)	4 (3.9)	13 (38.2)	< 0.0001
ECMO, n (%)	10 (9.8)	1 (2.94)	0.290
CHB required PPM, n (%)	3 (2.94)	1 (2.94)	1.00
Chylothorax, n (%)	9 (8.8)	7 (20.6)	0.065
Phrenic palsy, n (%)	2 (1.96)	1 (2.94)	0.736
Sepsis, n (%)	12 (11.8)	8 (23.5)	0.093
NEC, n (%)	3 (2.94)	4 (11.76)	0.044
AKI, n (%)	6 (5.9)	3 (8.82)	0.550
Peritoneal dialysis, n (%)	3 (2.94)	2 (5.9)	0.430
Stroke, n (%)	3 (2.94)	0	0.312
Intracranial haemorrhage, n (%)	6 (5.9)	5 (14.7)	0.102

ICU, intensive care unit; RACHS-1, risk-adjusted congenital heart surgery; ECMO, extracorporeal membrane oxygenation; CHB, complete heart block; PPM, permanent pacemaker; NEC, necrotizing enterocolitis; AKI, acute kidney injury

Overall, there was no significant difference in surgical outcomes between the first and second half of the 10-year period as shown in Table 6.

**Table 6:** Surgical outcomes between the first and second half of the 10-year period

Variables of outcome	From 2011 to 2015 N = 70	From 2016 to 2019 N = 66	*P* value	Remarks
Age, median (IQR)	17.5 (11–25.25)	20 (9–25.25)	0.922	Mann–Whitney U test
Weight, median (IQR)	3 (2.5–3.2)	3 (2.7–3.5)	0.210	Mann–Whitney U test
Length of stay in days, median (IQR)	22 (13–43)	26 (16.5–51)	0.157	Mann–Whitney U test
Mechanical ventilation in days, median (IQR)	6 (3–12.25)	7 (3.75–11)	0.725	Mann–Whitney U test
ICU stay, days, median (IQR)	15 (8–31)	20 (12–39)	0.056	Mann–Whitney U test
Mortality/discharge, n (%)	8 (11.4)/62 (88.6)	7 (10.6)/59 (89.4)	0.878	Chi-Square Tests

## Discussion

Surgical techniques of neonatal cardiac surgery evolved and were gradually standardized since the first pioneering neonatal surgeries performed on patients with TGA in the 1970s, allowing complex repairs and palliation for lesions such as HLHS that were previously considered inoperable [9]. With time, even symptomatic neonates with tetralogy of Fallot were repaired in the neonatal period rather than being subject to palliative shunts. Recent advances in the treatment of these patients, further improved results [10]. Choosing an ideal outcome measure for neonatal cardiac surgery for its ease of estimation, reproducibility, and, most importantly, independently remains a controversy. Many investigators use regular variables such as infection, lactate, and blood pressure, others have used surrogate outcome measures, such as low inotropic score and vasoactive inotropic score, and others postulate composite outcomes, which combine several components into a single measure [4–6]. In our study, we categorized the outcome into primary and secondary, the primary outcome was the operative mortality (in-hospital death) and secondary outcomes included hospital length of stay, ICU stay, duration of mechanical ventilation. The outcome measures we analyzed were those commonly used in the literature such as the requirement of ECMO support, early postoperative cardiac catheterization intervention, delayed chest closure, diaphragmatic paralysis, chylous effusion, arrhythmias, cerebral infarction, intracranial hemorrhage, limb ischemia, NEC, AKI, hepatic injury, and sepsis. Mortality remains a good measure of success in neonatal programs and an arena where the volume–quality relationship is most acutely observed [11]. We recorded an 11% overall operative mortality rate for all corrective and palliative neonatal cardiac surgeries. This compares favorably with results reported from common international centers [12, 13] and is slightly less than the operative mortality reported in the Society of Thoracic Surgeons (STS) database (12.2%) and the European Association for Cardio-Thoracic Surgery (EACT) database (13.3%) [14]. Nevertheless, it is a challenging task to compare outcomes and survival rates due to the complex relationship between cardiac surgical case volumes and mortality rates [15]. The report of cardiac surgery case volume in our study revealed a total of 1155 cardiac surgeries for CHD; of these, 136 (11.8%) were performed on neonates. As such, analysis by multivariable logistic regression identified three independent risk factors for mortality; these were the need for postoperative ECMO support [OR (95% CI) 0.147(0.022–0.983), *P* = 0.048], the occurrence of postoperative intracranial hemorrhage [OR (95% CI) 0.141(0.025–0.792), *P* = 0.026], and incidence of AKI [OR (95% CI) 0.114(0.015–0.849), *P* = 0.034], but the age at surgery, weight, prematurity, RACHS-1 scores, CPB, and other postoperative complications were not found to be independent predictors of operative mortality [16, 17]. These findings do not appear to be consistent with previous cohorts that reported age, weight, and RACHS-1 score are independent predictors of operative mortality but are consistent with other more recent studies [18, 19]. These conflicting results for the different centers illustrate what we have stated here regarding the selection of appropriate outcome measures in relation to the volume of cardiac surgeries and mortality rates. In contrast, analysis of risk factors for secondary outcomes such as hospital length of stay, ICU stay, duration of mechanical ventilation is easier to estimate, reproducible, and independent of risk factors for the primary outcome (operative mortality) [20]. We recorded the median postoperative duration of mechanical ventilation, ICU stay, and hospital length of stay of 6, 18, and 24 days, respectively. In this cohort, the patients with a history of prematurity, low birth weight, older age, or smaller bodyweight at the time of surgery had postoperative prolonged hospital length of stay. The higher-complexity operations and surgeries with CPB were associated with prolonged hospital length of stay. On the other hand, prolonged hospital length of stay was not significantly related to the requirement of ECMO support, CPB time, aortic cross-clamp time, and postoperative complications (apart from NEC). These results agree with most literature [8, 21, 22].

### Limitations

The limitations of this study include retrospective study design and small case volume. Additionally, as this was a single institutional study, the results need to be explored further. We focused only on short-term results, longer follow-up with neurological assessment and co-morbidities outcome would have been more informative.

## Conclusion

The early surgical outcomes of CHD achieved in neonates are encouraging. After neonatal cardiac surgery, age at surgery and weight may not be independent predictors of survival rate, while the need for postoperative ECMO support, postoperative intracranial hemorrhage, and acute kidney injury could have a significant association with mortality. Older age at surgery, smaller body weight, lower birth weight, prematurity, higher-complexity operations, need for CPB, and NEC were associated with prolonged hospital length of stay.

## Data Availability

The datasets used and/or analyzed during the current study are available from the corresponding author on reasonable request.

## Abbreviations

D-TGA: Dextro transposition of great arteries
PDA: Patent ductus arteriosus
HLH: Hypoplastic left heart syndrome
TAPVR: Total anomalous pulmonary venous return
DORV: Double outlet right ventricle
VSD: Ventricular septal defect
ALCAPA: Aberrant left coronary artery from the pulmonary artery
RACHS-1: Risk Adjustment for Congenital Heart Surgery
COA: Coarctation of the aorta
MBTS: Modified Blalock Taussig Shunt
ECMO: Extracorporeal membrane oxygenation
ICU: Intensive care unit
PPM: Permanent pacemaker
CHB: Complete heart block
NEC: Necrotizing enterocolitis
AKI: Acute kidney injury

## References

[1] Khairy P, Ionescu-Ittu R, Mackie AS, Abrahamowicz M, Pilote L, Marelli AJ (2010). Changing mortality in congenital heart disease. J Am Coll Cardiol.

[2] Hovels-Gurich HH, Konrad K, Wiesner M, Minkenberg R, Herpertz-Dahlmann B, Messmer BJ (2002). Long term behavioural outcome after neonatal arterial switch operation for transposition of the great arteries. Arch Dis Child.

[3] Ballweg JA, Wernovsky G, Gaynor JW (2007). Neurodevelopmental outcomes following congenital heart surgery. Pediatr Cardiol.

[4] Wernovsky G, Wypij D, Jonas RA, Mayer JE, Hanley FL, Hickey PR (1995). Postoperative course and hemodynamic profile after the arterial switch operation in neonates and infants: a comparison of low-flow cardiopulmonary bypass and circulatory arrest. Circulation.

[5] Gaies MG, Gurney JG, Yen AH, Napoli ML, Gajarski RJ, Ohye RG (2010). Vasoactive-inotropic score as a predictor of morbidity and mortality in infants after cardiopulmonary bypass. Pediatr Crit Care Med.

[6] Hoffman TM, Wernovsky G, Atz AM, Kulik TJ, Nelson DP, Chang AC (2003). Efficacy and safety of milrinone in preventing low cardiac output syndrome in infants and children after corrective surgery for congenital heart disease. Circulation.

[7] Jenkins KJ, Gauvreau K, Newburger JW, Spray TL, Moller JH, Iezzoni LI (2002). Consensus-based method for risk adjustment for surgery for congenital heart disease. J Thorac Cardiovasc Surg.

[8] Butts RJ, Scheurer MA, Zyblewski SC, Wahlquist AE, Nietert PJ, Bradley SM, Atz AM, Graham EM (2014). A composite outcome for neonatal cardiac surgery research. J Thorac Cardiovasc Surg.

[9] Jatene AD, Fontes VF, Paulista PP (1976). Anatomic correction of transposition of the great vessels. J Thorac Cardiovasc Surg.

[10] Tweddell JS (2016). Advances in neonatal cardiac surgery: recent advances, the lowhanging fruit, what is on the horizon and the next moonshot. Curr Opin Cardiol.

[11] Padley JR, Cole AD, Pye VE, Chard RB, Nicholson IA, Jacobe S, Baines D, Badawi N, Walker K, Scarfe G, Leclair K, Sholler GF, Winlaw DS (2011). Five-year analysis of operative mortality and neonatal outcomes in congenital heart disease. Heart Lung Circ.

[12] Bove T, Francois K, De Groote K, Suys B, De Wolf D, Verhaaren H (2004). Outcome analysis of major cardiac operations in low weight neonates. Ann Thorac Surg.

[13] Shen I, Giacomuzzi C, Ungerleider RM (2003). Current strategies for optimizing the use of cardiopulmonary bypass in neonates and infants. Ann Thorac Surg.

[14] Jacobs JP, Jacobs ML, Maruszewski B (2005). Current status of the European Association for Cardio-Thoracic Surgery and the Society of Thoracic Surgeons Congenital Heart Surgery Database. Ann Thorac Surg.

[15] Welke KF, O’Brien SM, Peterson ED, Ungerleider RM, Jacobs ML, Jacobs JP (2009). The complex relationship between pediatric cardiac surgical case volumes and mortality rates in a national clinical database. J Thorac Cardiovasc Surg.

[16] Jenkins KJ, Gauvreau K, Newburger JW, Spray TL, Moller JH, Iezoni LI (2002). Consensus-based method for risk adjustment for surgery for congenital heart disease. J Thorac Cardiovasc Surg.

[17] Kang N, Cole T, Tsang V, Elliott M, de Leval M (2004). Risk stratification in paediatric open-heart surgery. Eur J Cardiothorac Surg.

[18] ElMahrouk AF, Ismail MF, Hamouda T, Shaikh R, Mahmoud A, Shihata MS, Alradi O, Jamjoom A (2019). Extracorporeal membrane oxygenation in postcardiotomy pediatric patients-15 years of experience outside Europe and North America. Thorac Cardiovasc Surg.

[19] Al-Radi OO (2020). Are neonatal age and small weight predictive of in-hospital death and prolonged hospital stay in children undergoing heart surgery?. Cardiothorac Surg.

[20] Curzon CL, Milford-Beland S, Li JS, O'Brien SM, Jacobs JP, Jacobs ML, Welke KF, Lodge AJ, Peterson ED, Jaggers J (2008). Cardiac surgery in infants with low birth weight is associated with increased mortality: analysis of the Society of Thoracic Surgeons Congenital Heart Database. J Thorac Cardiovasc Surg.

[21] Bakshi KD, Vaidyanathan B, Sundaram KR, Roth SJ, Shivaprakasha K, Rao SG, Nair SG, Chengode S, Kumar RK (2007). Determinants of early outcome after neonatal cardiac surgery in a developing country. J Thorac Cardiovasc Surg.

[22] Hasegawa T, Masuda M, Okumura M, Arai H, Kobayashi J, Saiki Y, Tanemoto K, Nishida H, Motomura N (2017). Trends and outcomes in neonatal cardiac surgery for congenital heart disease in Japan from 1996 to 2010. Eur J Cardiothorac Surg.

